# The oxytocin paradox

**DOI:** 10.3389/fnbeh.2014.00048

**Published:** 2014-02-17

**Authors:** Richard A. I. Bethlehem, Simon Baron-Cohen, Jack van Honk, Bonnie Auyeung, Peter A. Bos

**Affiliations:** ^1^Department of Psychiatry, Autism Research Centre, University of CambridgeCambridge, UK; ^2^Cambridgeshire and Peterborough NHS Foundation Trust, CLASS ClinicCambridge, UK; ^3^Department of Experimental Psychology, Utrecht UniversityUtrecht, Netherlands; ^4^Department of Psychiatry and Mental Health, J-Block Groote Schuur Hospital Observatory, University of Cape TownCape Town, South Africa; ^5^Psychology Department, Edinburgh UniversityEdinburgh, UK

**Keywords:** oxytocin, reward processing, anxiety, autism spectrum disorder, translational research, reward

In 2005, Kosfeld et al. published their now seminal paper showing that intranasal oxytocin (OXT) administration increased interpersonal trust (Kosfeld et al., 2005). This finding spawned broad interest into the effects of OXT on social and emotional behavior in humans (Bos et al., 2012), and its implications for translational medicine (Meyer-Lindenberg et al., 2011; Striepens et al., 2011). Over the years OXT has gained the reputation of facilitating empathy and affiliation, based on early findings reporting beneficial effects of OXT on trust (Kosfeld et al., 2005), social support (Heinrichs et al., 2003), and processing social information (Hollander et al., 2007; Savaskan et al., 2008; Unkelbach et al., 2008; Hurlemann et al., 2010). This view is supported by studies showing that OXT improves cognitive empathic abilities such as mindreading (Domes et al., 2007; Bartz et al., 2010; Guastella et al., 2010) and recognizing positive emotional expressions (Marsh et al., 2010). As a result of these positive effects on social behavior, there has been considerable speculation about OXT's therapeutic potential in people with social and emotional disabilities.

This prosocial view of OXT has been challenged by findings showing that the effects of OXT are strongly context-dependent (Bartz et al., 2011; Bos et al., 2012). For example, OXT has also been shown to increase envy and gloating (Shamay-Tsoory et al., 2009), defensiveness toward out-group members (De Dreu et al., 2010, 2011) and increased in-group conformity (Stallen et al., 2012). Although this ethnocentrism might be considered prosocial within one's own group, defensiveness toward an out-group is not, and extreme ethnocentrism often leads to nationalism or even racism.

This paradox gives rise to two questions. First, how can the beneficial effects of OXT on empathy, trust and affiliation be compatible with its seemingly contradictory anti-social effects? Second, what implication does this have for the therapeutic potential of OXT in social and emotional neurodevelopmental conditions? With regard to the first question, we provide a brief overview of the literature aiming to understand the mechanism(s) underlying OXT's efficacy. There are two main perspectives on how OXT affects social behavior (via *anxiety reduction* or *increasing social salience*), which try to reconcile its pro- and anti-social roles, but there have been no attempts to date to integrate these into a single viewpoint. A potential third factor, *reward sensitivity* might aid this integration. We propose a model that unites these perspectives, providing new avenues of research into OXT's efficacy in social and emotional neurodevelopmental conditions.

## Mechanisms underlying OXT's effect on social behavior

Early attempts to reconcile different effects of OXT on social behavior suggested interaction effects between stress, anxiety and social approach (Heinrichs and Domes, 2008; Heinrichs et al., 2009). An excellent review (Churchland and Winkielman, 2012) highlights OXT's anxiolytic effects as a key mechanism. Churchland and Winkielman (2012) argue that low-level anxiety drives higher-order social-cognitive effects. A large body of animal literature also supports the idea of OXT as an anxiolytic (Bale et al., 2001; Ring et al., 2006; Ebitz et al., 2013). Ebitz and colleagues showed that OXT reduces social vigilance in the rhesus macaque (Ebitz et al., 2013), potentially by reducing social threat or anxiety. In humans, a reduction in anxiety could facilitate “pro-social” behavior by eliminating social threat during social interactions. Consistent with this, there is evidence for anxiolytic effects of OXT in humans with anxiety disorder (Labuschagne et al., 2010). Labuschagne et al. (2010) showed that OXT decreases amygdala activity in response to fearful faces in people with a generalized social anxiety disorder (GSAD), to the level of typical controls.

Decreased social threat following OXT administration is also in line with findings showing improved coping mechanisms in stressful situations (Heinrichs et al., 2003). Although there is broad consensus about the anxiolytic properties of OXT, its antisocial effects are not entirely explained by this. As mentioned above, earlier findings (De Dreu et al., 2010, 2011) demonstrated increased in- out-group differences after OXT administration (but see Shamay-Tsoory et al., 2013). However, reduced anxiety would be expected to lead to decreased in- and out-group differences by also reducing anxiety toward out-group members. In addition, direct empirical tests of the anxiolytic effects of OXT in humans has shown that oxytocin can increase anxiety toward an unpredictable threat (Grillon et al., 2012). This suggests that OXT's anxiolytic properties cannot completely account for the differences in behavioral findings.

Another perspective on the effects of OXT on social behavior involves social salience. It has been hypothesized that OXT increases sensitivity for salient social cues (Bartz et al., 2011; Striepens et al., 2012; Wittfoth-Schardt et al., 2012). This is known as the “social salience hypothesis” and fits with findings of improved mind-reading and increased eye contact (Domes et al., 2007; Guastella et al., 2008). Increasing the salience of subtle contextual eye-cues could indeed improve mind-reading and might cause an attentional bias toward such cues. However, the effects of OXT administration on promoting ethnocentrism emerge, regardless of the valence of an intergroup comparison (De Dreu et al., 2010, 2011). This suggests that these effects cannot be explained by the notion that OXT administration generally increases perceptual salience for social cues, as that would alter valence ratings. Interestingly, recent work has demonstrated that affiliative emotion (a primary target in OXT research) may be distinguishable from general emotional valence (Moll et al., 2012). In addition, Striepens et al. (2012) showed that OXT administration did not alter valence ratings for aversive social stimuli. Since the administration of OXT also facilitated startle response and memory for negative cues, these authors conclude that OXT administration may promote general “approach and protective behavior, but with heightened caution” (Striepens et al., 2012). It is likely that OXT does affect affiliative emotion but it is possible that, depending on the context, general emotional valence is not affected. How OXT does this remains an open question. It seems that, by itself, the social salience hypothesis also cannot fully explain the varied behavioral findings.

In addition to being an anxiolytic and potentially increasing perceptual salience of social cues, OXT may exert its effects on social behavior via a third mechanism: increased reward sensitivity (Leckman, 2011; Strathearn, 2011; Dolen et al., 2013). The idea that reward sensitivity is an important contributing factor to OXT's effect on social behavior is not new (Young et al., 2001), nor necessarily incompatible with the social salience hypothesis (Weisman and Feldman, 2013). Animal studies have shown strong links between OXT and the reward circuitry, specifically in the ventral striatum (Shahrokh et al., 2010; D'Cunha et al., 2011; Keebaugh and Young, 2011; Baracz and Cornish, 2013). A recent study also demonstrated involvement of reward circuits in the efficacy of OXT in humans (Groppe et al., 2013). Groppe et al. (2013) showed that the modulation of social salience by OXT takes place in the human ventral tegmental area (a key area in the mesocorticolimbic dopamine-system), confirming previous animal findings (Shahrokh et al., 2010). Similarly, Strathearn et al. (2009) showed that the ventral striatum is activated during mother-infant interaction, which is accompanied by increased peripheral OXT release.

Two neurobiological pathways may underlie OXT's effect on reward processing: an OXT-dopamine (Shahrokh et al., 2010; Baracz and Cornish, 2013) pathway and an OXT-opioid pathway (Gu and Yu, 2007). The former likely influences the “wanting” aspect of reward processing, whereas the latter likely pertains to the “liking” aspect of reward processing (Berridge et al., 2009). The opioid-OXT pathway is also likely to be involved in OXT's effects on social bonding and affiliation (Burkett and Young, 2012). If either type of reward processing is affected by OXT it is possible that this will also affect reward learning (Berridge et al., 2009).

Lastly, OXT has also been repeatedly associated with drugs of abuse and addiction (Burkett and Young, 2012; Carson et al., 2013; Sarnyai and Kovacs, 2013), which also act on the reward system. It has even been suggested that OXT's facilitation of social reward sensitivity may somewhat override that of drug-induced reward (Sarnyai and Kovacs, 2013; Tops et al., 2013). The way in which reward sensitivity precisely affects social interactions remains unknown, but these findings suggest that OXT influences the means by which social reward sensitivity modulates social behavior. Additionally, context may play a role in the exact type of pathway affected.

## Future directions

**Figure 1:**
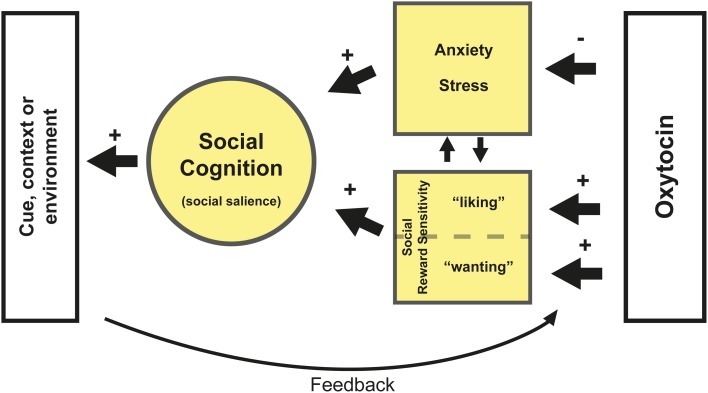
**Oxytocin reduces anxiety and stress for social interaction and increases social reward sensitivity.** With respect to the latter, OXT likely affects hedonistic reward processing (e.g., “liking”) via its interaction with the opioid system and incentive reward processing (e.g., “wanting”) via a striatal-dopamine pathway (Berridge et al., 2009). Which pathway is affected likely depends on the specific context. Both reduced anxiety and increased reward sensitivity might increase sensitivity for social salience but also directly improve aspects of social cognition depending on person and context. Furthermore the subsequent contextual or environmental feedback affects the sensitivity and plasticity of the OXT system depending on its valence (e.g., positive or negative feedback). Positive feedback after OXT administration, such as pleasant social interaction, might reinforce the sensitivity for social reward and further decrease anxiety. This feedback potentially also alters the plasticity of the OXT system. For example, more OXT may be released during social interaction. Another potential scenario is that OXT administration leads to decreased anxiety, which in turn leads to heightened social salience. Which may also involve paying more attention to (potentially) negative social cues. Administration of OXT might therefore have a stronger impact on this negative feedback in neurotypical individuals, as was shown by Striepens et al. (2012). Lastly, any type of feedback resulting from altered social reward processing is likely to affect reward learning.

The three different mechanisms reviewed here are not mutually exclusive. For example, decreased anxiety could lead to increased sensitivity for social salience, and vice versa. Together, anxiety and social reward sensitivity influence attribution of social salience. They might, in a given context, either promote or impede social behavior. We hypothesize that the mechanism by which OXT affects social behavior depends upon these two systems (see Figure 1). Subsequently, the environment provides feedback to the OXT system about the resulting social behavior, influencing anxiety and reward sensitivity. This synergy in turn determines the salience of social cues in a particular context.

In people with autism spectrum conditions (ASC) or GSAD this system might be disturbed, resulting in social interaction deficits. Recent research has shown hypoactivation in the reward circuitry in ASC during social reward processing (Delmonte et al., 2012). In addition, people with ASC show increased anxiety (White et al., 2009), which might lead to an attentional bias toward negative cues (Winton et al., 1995) in social interaction. Together, these factors create a negative feedback loop, which further enhances this negative spiral. In such situations OXT may have a positive effect by reducing anxiety and increasing the rewarding value of social interactions. The social motivation hypothesis (SMH) for ASC (Stavropoulos and Carver, 2013) indeed suggests that reward sensitivity is decreased in ASC and that OXT helps alleviate this. For example, it has been shown that OXT administration leads people with ASC to respond more appropriately to reciprocity in a cyberball game (Andari et al., 2010). Increased reward sensitivity would thus enable participants to better distinguish which player offers the most positive interaction. Furthermore, reduced anxiety for social interaction (such as direct eye contact) may modulate gaze-time (Domes et al., 2013d), and increased reward sensitivity may shift attention toward positive social cues (Domes et al., 2013c). In individuals with ASC, this could modulate face and emotion processing (Domes et al., 2013a,b). Reducing anxiety and improving the reward value of social interaction would also improve emotion recognition and social behavior in individuals with and without ASC (Hollander et al., 2007; Guastella et al., 2010; Domes et al., 2013b). Although the anxiolytic effects of OXT have been widely researched, its combination with reward sensitivity may prove to be a novel target for OXT administration in ASC. Interestingly, this might also help explain the variation of the effects of OXT seen across the population.

In individuals that do not experience heightened anxiety in social situations or experience reduced reward sensitivity, OXT might not necessarily show beneficial effects. Moreover, it might even impede social behavior. For example, by decreasing anxiety social vigilance might become blunted (Ebitz et al., 2013), while increasing reward sensitivity could possibly lead to gloating (Shamay-Tsoory et al., 2009). Furthermore, ethnocentrism occurs naturally in the normal population (Yzerbyt and Demoulin, 2010): under normal circumstances people tend to favor their own group over out-group members. Thus, interaction with in-group members is generally considered more rewarding. Also, a negative interaction with out-group members may constitute a form of negative feedback compared to positive interaction with in-group members. Our model predicts that administration of OXT would increase these differences. It strengthens the rewarding type of interaction and reinforces the effect of negative feedback. In this context, OXT indeed promotes ethnocentrism (De Dreu et al., 2010, 2011).

In conclusion, over recent years it has become clear that the effects of OXT administration on human social behavior are not best framed in absolute terms such as pro- or antisocial. We aim to shift the focus to underlying core processes such as anxiety and reward sensitivity. When applied to studied contexts, the modulation of OXT on these core processes can lead to different behavioral outcomes dependent on person and situation, especially since reward processing and anxiety in specific contexts differs from person to person. More research is needed to investigate how the dynamics of OXT modulation on these core processes translates to different contexts and groups.
